# The Impact of Adjunct Medical Therapy on Survival after Spine Metastasis: A Systematic Review and Pooled Data Analysis

**DOI:** 10.3390/cancers16071425

**Published:** 2024-04-07

**Authors:** Lilly Groszman, Jonathan A. Hubermann, Paul Kooner, Nawaf Alamiri, Anthony Bozzo, Ahmed Aoude

**Affiliations:** Department of Orthopaedic Surgery, McGill University Health Centre, Montreal, QC H4A 3J1, Canada; lilly.groszman@mail.mcgill.ca (L.G.); jonathan.hubermann@mail.mcgill.ca (J.A.H.); nawaf.alamiri@mail.mcgill.ca (N.A.);

**Keywords:** targeted therapy, metastatic spinal tumors, personalized treatment approaches

## Abstract

**Simple Summary:**

In recent years, targeted therapy has significantly improved the lives of patients suffering from spine metastasis. However, traditional scoring systems used to predict treatment outcomes may not reflect these advancements. This study aims to explore the latest literature on medical therapy options for metastatic spinal tumors, particularly focusing on targeted therapy compared to other treatments. Through a systematic review and data analysis, this research highlights the effectiveness of targeted therapy, especially in lung and breast cancers, in prolonging the median overall survival. By considering the recent advances in medical oncology, these findings emphasize the importance of incorporating personalized treatment approaches into the management of metastatic spine tumors. This study provides valuable insights for clinicians and researchers in optimizing treatment strategies for patients with spinal metastases.

**Abstract:**

Targeted therapy has greatly improved the outlook for patients with spinal metastatic cancers. Scoring systems like the Tokuhashi or Tomita scores are commonly used to predict prognosis and inform surgical decisions, but they are outdated and fail to consider recent advancements. We aimed to investigate the current state of the literature and treatment options pertaining to advancements in targeted therapy compared to other forms of medical management for metastatic spinal tumors. This study represents the first comprehensive systematic review that encompasses the most common primary cancers that metastasize to the spine and evaluates the median overall survival (mOS) across five different medical treatment modalities as well as surgical intervention. Additionally, our study analyzes the tumor receptor status in conjunction with these treatments. A PubMed search was conducted, and according to the PRISMA guidelines, 28 articles out of 1834 met the inclusion criteria. The pooled data analysis highlighted the superior efficacy of targeted therapy, evidenced by a significant improvement in the mOS and lower hazard ratios in patients with lung and breast cancers who received targeted therapy compared to those who did not. Our study provides valuable insights into the recent advancements in the medical management of metastatic spinal tumors. Future indications include incorporating this literature into personalized treatment approaches for metastatic spinal tumors.

## 1. Introduction

The number of patients with metastatic spine disease continues to rise worldwide [1]. Thoroughly evaluating the prognosis prior to treating a metastatic spine tumor holds paramount significance in guiding treatment options. Numerous scoring systems and treatment algorithms were initially introduced during a time when systemic oncological treatments for metastatic spinal tumors were limited. These scoring systems have since been widely embraced as effective and straightforward tools to guide surgical management [2]. Scoring systems such as the Tokuhashi or Tomita scores are often used to prognosticate the life expectancy of a patient and ultimately guide treatment and surgical management [3]. However, the medical landscape has evolved with the aggressive treatment of metastatic tumors, rendering these scoring systems outdated. For example, existing algorithms that categorize stage four lung cancer as having a poor prognosis in the context of spinal metastases may be outdated due to the emergence of specific lung cancer subtypes, immunotherapies, and targeted therapies (TTs) [4]. In a previous study, Aoude et al. [5] demonstrated the effectiveness of the updated Tokuhashi score in distinguishing patients with poor prognoses from those with moderate to good prognoses. However, although the revised Tokuhashi score can be utilized to estimate patient survival, its application should be approached with caution and might necessitate additional modifications. Adjusting the score to reflect population-specific characteristics could enhance its accuracy in identifying patients with spinal metastases who would benefit from surgical intervention [6]. With the emergence of tumor molecular sequencing and genetic profiling, current prognostic algorithms should reflect the significant advancements in targeted systemic therapy [7,8]. Chen et al. [9] and Morgen et al. [10] demonstrated that patients with spinal metastases from certain cancer types have improved prognoses due to rapid treatment advancements in immunotherapy. Furthermore, even with advancements in en bloc surgical resection and aggressive radiation therapies, controlling metastatic spinal disease often remains challenging. The support of adjunct medical therapy is crucial as it plays a vital role in targeting residual microscopic disease and preventing recurrence [1]. This underscores the importance of considering these advancements in prognosticating the overall survival for various cancers that metastasize to the spine [11].

The primary aim of this research is to consolidate evidence for approaches to treat metastatic spinal tumors and guide the decision-making process by investigating the current state of the literature and treatment options pertaining to advancements in targeted systemic therapy compared to conventional management for metastatic spinal tumors. This study represents the first comprehensive systematic review that encompasses the most common primary cancers that metastasize to the spine while examining five distinct medical treatment modalities in conjunction with the analysis of the tumor receptor status.

## 2. Materials and Methods

### 2.1. Electronic Database Search Strategy

**Figure 1:**
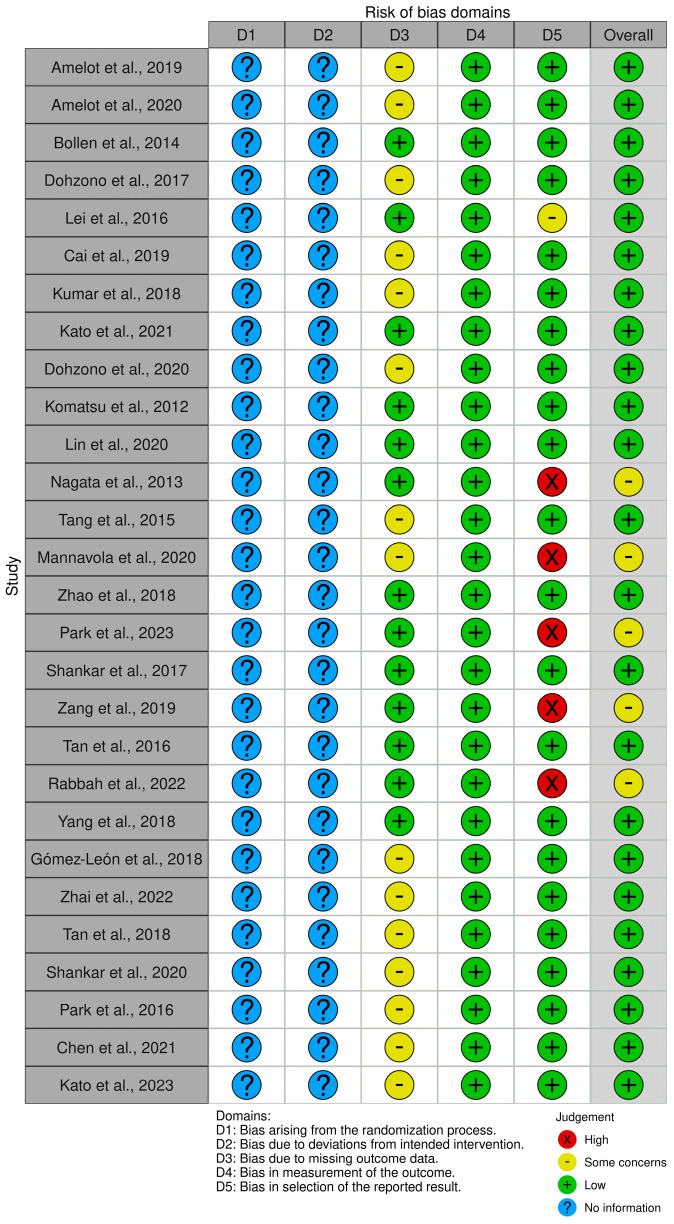
Risk of bias assessment of included studies [11,12,13,14,15,16,17,18,19,20,21,22,23,24,25,26,27,28,29,30,31,32,33,34,35,36,37,38].

We conducted a systematic review of the existing literature that addresses the clinical and molecular factors linked to survival rates in patients with spinal metastatic disease. This systematic review was conducted according to the Preferred Reporting Items for Systematic Reviews and Meta-Analyses (PRISMA) [12] guidelines, and bias was assessed using the risk of bias assessment tool outlined by the Cochrane review [13] (Figure 1). The protocol was not registered. The selection process is detailed in Figure S1, and the PRISMA checklist can be found in Table S1. In August 2023, study selection was carried out by two authors, with the aid of a librarian, who searched the PubMed database to identify articles for review. Eight broad search strategies were used for each type of primary tumor that metastasizes to the spine (breast, lung, melanoma, thyroid, gastric, osteosarcoma, and prostate tumors). A combination of keywords and MeSH terms were used (see Appendix A for the full search strings that were used for data extraction).

### 2.2. Exclusion and Inclusion Criteria

Two authors independently reviewed all abstracts and selected them for detailed assessments of the full articles. Due to advancements in targeted receptor therapy regimens, only English articles published in a peer-reviewed, PubMed-indexed journal after January 2005 and until August 2023 were considered for review. Table 1 describes the inclusion and exclusion criteria of this study. For inclusion, patients must have a primary diagnosis of one of the eight most common primary tumors that metastasize to spine, the publication must present an original article reporting a case series greater than five patients, and it must report on the type of treatment performed. All publications with insufficient information of the type of treatment performed and associated outcomes were excluded.

### 2.3. Data Extraction

Two authors extracted and summarized the data from the included studies as follows: study demographics (year of publication, study period, design, multicenter study, and LoE) (Table 2), participant demographics (number of participants, age, and sex), primary tumor location, treatment type, survivorship, tumor receptor status, and primary tumor histology.

### 2.4. Statistical Methods

We employed a tiered approach for the data analysis. Initially, we identified the variables reported in each study for every treatment type–primary site combination. A pooled mOS was calculated in months as a weighted median of medians, with the weights proportional to the sample sizes, which were normalized to sum to 1, akin to a fixed-effect analysis. The 95% confidence intervals were approximated using the weighted quantiles [42]. Studies were only selected for inclusion in this pooled analysis of survival if they provided the number of patients (n) and mOS for both the treatment and control groups (Table 3). For the meta-analysis of hazard ratios (HRs), we applied the generic inverse-variance random effects model, which incorporated the multivariate Cox proportional hazards regression model’s hazard ratios and their confidence intervals [43]. Heterogeneity among studies was quantified using the DerSimonian–Laird estimator [44,45]. No meta-analysis was performed in cases where only a single study reported the applicable measures for a treatment type–primary site subgroup. This criterion was established to avoid skewing the results with non-representative data. Kaplan–Meier survivorship curves were generated for the lung cancer primary site group, and survival was compared between the subgroup that underwent targeted therapy and the group that did not receive this treatment. All analyses were conducted using R software, version 4.3.1 (R Foundation for Statistical Computing, Vienna, Austria). A *p*-value < 0.05 was set as the threshold for statistical significance.

## 3. Results

### 3.1. Study Selection

In total, 1834 studies were identified through the literature search. Two authors independently screened the titles and abstracts based on the PRISMA guidelines and by using the study’s inclusion and exclusion criteria. Of these, there were 219 duplicates and 1475 studies marked as irrelevant. A total of 140 studies were then assessed for full-text reviews; 112 of these studies were excluded for study design, outcomes, patient population, language, intervention, and comparators. There was a total of 28 studies remaining for inclusion. Any disagreement was resolved by the senior author after discussion and consensus.

### 3.2. Study Demographics

A summary of the demographics of the chosen studies included is shown in Table 2. Out of the 28 papers included in the analysis, 16 focused on lung cancer, 5 on breast cancer, 3 on renal cancer, 3 on melanoma, and 1 on thyroid cancer, all with spinal metastases. None of the papers on primary gastric cancer, prostate cancer, or bone cancer met our inclusion criteria for review. All data were based on retrospective studies with level III or level IV quality of evidence and a low to moderate risk of bias (Figure 1). Only six of the included studies were multicenter studies.

### 3.3. Patient Demographics

A comprehensive evaluation of 3238 patients was conducted, with patients with lung cancer forming the largest group, totaling 2137 individuals. This group included mostly non-small-cell lung cancer (NSCLC) subtypes (n = 2020), with a total weighted mean age of 58.3 years (Table 4). The gender distribution was notably skewed towards males in the lung cancer studies, accounting for 61% of all patients. The breast cancer studies included a smaller cohort of 591 patients, with a weighted mean age of 58.3 years, and a minimal male representation (only 0.5%). Other cancer types, such as renal, melanoma, and thyroid, were also represented, albeit in smaller numbers, contributing to the demographic diversity of our studied patient cohort.

### 3.4. Median Overall Survival (mOS)

The concept of mOS is the most common measure used to report outcomes of oncology clinical trials [46]. We utilized the mOS as the primary outcome measure. The lung cancer studies revealed a pooled mOS of 6.7 months (95% CI: 4.8–11.6) among all patients, possibly reflecting the cancer’s aggressiveness and detection difficulties [47]. The breast cancer studies showed a longer mOS of 28.3 months (95% CI: 18.0–159.8). Renal cancer had an even higher mOS at 58.3 months (95% CI: 23.6–100.0), and the melanoma studies reported an mOS of 10.6 months (95% CI: 3.9–18.0). Finally, a single thyroid cancer study indicated a high mOS of 123.0 months.

### 3.5. Impact of Medical Therapy on mOS

Section 3.5.1, Section 3.5.2, Section 3.5.3, Section 3.5.4, Section 3.5.5 describes the impact of targeted therapy, chemotherapy, radiation therapy, immunotherapy, and bisphosphonate therapy on mOS for patients with metastases to the spine (Table 5).

#### 3.5.1. Targeted Therapy (TT)

**Figure 2:**
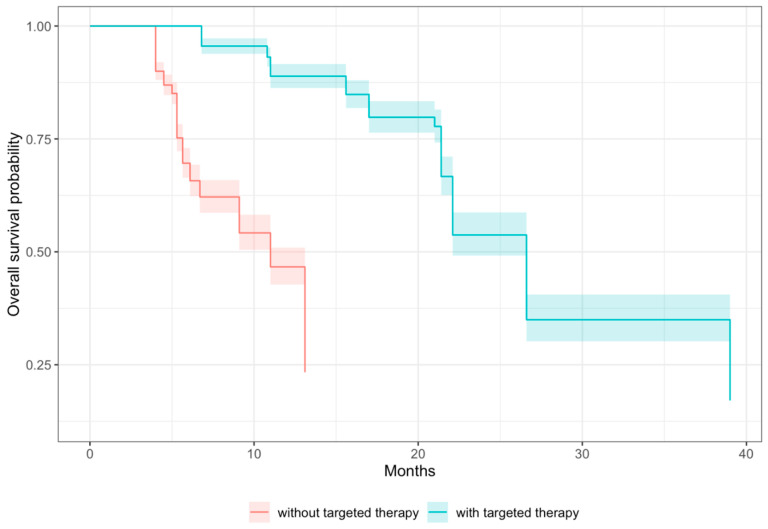
Kaplan–Meier survivorship curves for lung cancer subgroups treated with and without targeted therapy.

Most patients with lung cancer in the included studies received TT (15/16 studies reported isolated TT use), primarily epidermal growth factor receptor tyrosine kinase inhibitors (EGFR-TKI). Of these, 11 studies reported on the mOS. There was a marked improvement in the mOS for patients undergoing TT, with a pooled mOS of 21.4 months (95% CI: 11.0–23.6) compared to 5.7 months (95% CI: 4.0–10.9) for those who did not receive TT. Six studies specifically reported the univariate HRs for TT in patients with lung cancer with spinal metastases, each demonstrating favorable outcomes with statistical significance (*p* < 0.05). Furthermore, the pooled multivariate HR was 0.395 (95% CI: 0.296–0.527, *p* < 0.0001) across the studies, indicating that those who received TT had a 60.5% reduction in the risk of mortality compared to those who did not undergo TT, as illustrated by the Kaplan–Meier survivorship curves in Figure 2. For breast cancer, across three studies [27,30,37], 191 patients received TT, and 166 patients did not receive TT. Two of the studies [28,33] reported the mOS comparing those receiving TT (pooled mOS of 83.2 months (95% CI: 53.0–94.2)) and those without TT (pooled mOS of 32.9 months (95% CI: 25.0–54.3)). For the melanoma subgroup, two papers reported a univariate HR for TT use [27,30], with one [27] reporting a statistically significant HR of 0.24 (95% CI: 0.10–0.57, *p* < 0.0001).

#### 3.5.2. Chemotherapy

In our review, 11 of the lung cancer studies reported on outcomes for patients receiving isolated chemotherapy. Of the five papers that reported the mOS rates for both the treated and untreated groups, four studies indicated a higher mOS with chemotherapy, with one [34] showing statistical significance (*p* < 0.001). The remaining study [22] reported a lower mOS with chemotherapy, which was also statistically significant (*p* < 0.05). Two studies conducted a univariate analysis, both showing HRs < 1, suggesting improved prognosis with chemotherapy, with one [20] being significant (*p* < 0.001). In multivariate Cox regression analyses across three papers, two found significant HRs < 1 (*p* < 0.01) [20,40], while the third reported a non-significant HR > 1 [22]. The pooled mOS for patients with lung cancer receiving chemotherapy (n = 598) was 14.2 months (95% CI: 5.5–19.9) compared to 8.5 months (95% CI: 4.6–11.0) for those who did not receive chemotherapy (n = 721). The pooled HR was 0.604 (95% CI: 0.248–1.467) across the studies, indicating that those who received chemotherapy had a 39.6% reduction in the risk of mortality compared to those who did not undergo such treatment (*p* = 0.265). For breast cancer, only one paper reported on chemotherapy, revealing a significant improvement in the mOS (46 vs. 22 months, *p* = 0.013), with a multivariate HR of 0.312 (*p* = 0.003) [28]. Studies on renal, melanoma, and thyroid cancers either did not report on the use of chemotherapy or did not provide significant outcomes.

#### 3.5.3. Radiation Therapy (RT)

For lung cancer, 11 studies reported isolated RT use. Among these, three studies shared data on the mOS. Two of these studies found that patients treated with RT had a significantly higher mOS (*p* < 0.01) [26,34], while one reported a lower mOS with RT (*p* = 0.24) [15]. Three studies calculated the HRs, all indicating a reduced mortality risk with RT (HR < 1). The pooled mOS for patients with lung cancer who received RT (n = 740) was 13.5 months (95% CI: 5.1–22.9), while it was 12.2 months (95% CI: 6.5–15.2) for those who did not receive RT (n = 618). However, only one study, which conducted a multivariate analysis, found a hazard ratio reflecting a significant benefit (HR = 0.37, *p* < 0.01) [26]. All five breast cancer studies reported RT use. Two studies [14,37] presented mOS data (pooled mOS for RT: 45.8 months (95% CI: 45.7–46.0) compared to a pooled mOS for no RT: 33.5 months (95% CI: 29.0–39.6)). One reported an HR of 1.31 with RT, suggesting a higher mortality risk (*p* = 0.392) [37]. Regarding melanoma, one study showed a slight improvement in the mOS with RT (10.4 vs. 9.2 months), but without a significant *p*-value [14]. The HR indicated a slightly increased mortality risk with RT (1.24), but this was also not significant (*p* = 0.16). Finally, for thyroid cancer, a single study found a substantial increase in the mOS with RT vs. none (123.6 vs. 76.8 months, *p* = 0.497) [41].

#### 3.5.4. Immunotherapy

For lung cancer, only one study focused on the isolated use of immunotherapy, revealing an HR of 0.585, indicating a 41.5% reduction in mortality likelihood with immunotherapy (*p* = 0.095) [15]. In the context of breast and renal cancers, only one paper for each cancer type reported on the isolated use of immunotherapy, but neither provided detailed outcome data beyond the number of participants [21,37]. For melanoma, two papers addressed immunotherapy use. One of these [27] did not provide specific data on immunotherapy use alone but reported that combining immunotherapy with targeted therapy was significantly more effective than chemotherapy (HR-UV = 0.24, *p* < 0.0001; HR-MV = 0.32, *p* = 0.0002). Combining radiation therapy with immunotherapy was shown to be more beneficial than combining radiation therapy with targeted therapy (HR-MV = 0.50, *p* = 0.013). The other study [30] reported an mOS of 3.22 months with immunotherapy compared to 10.36 months without it. The univariate HR was 3.45 (*p* = 0.05), suggesting a higher mortality risk with immunotherapy.

#### 3.5.5. Bisphosphonate Therapy

Bisphosphonate therapy was utilized in only five studies, spanning four distinct cancer sites, namely lung, breast, melanoma, and thyroid, with a total of 296 patients across all studies. Four studies reported that patients receiving bisphosphonate therapy experienced a longer mOS compared to those who did not receive this treatment. In three of these studies, the prolongation of the mOS associated with bisphosphonate therapy was statistically significant (*p* < 0.05). The pooled HR for patients with lung cancer receiving treatment was 0.933 (95% CI: 0.579–1.503, *p* = 0.776).

### 3.6. Impact of Receptor Status on mOS 

For lung cancer, patients with EFGR+ status (n = 367) showed an mOS of 24.9 months (95% CI: 23.9–25.3) with a 33.6% reduction in the risk of mortality compared to those with EGFR-status (n = 467). Not enough data were reported on the ALK status to calculate a pooled mOS or HR. For breast cancer, patients with Basal (triple-negative) receptor status (n = 88) reported the lowest pooled mOS of 11.6 months (95% CI: 5.5–17.3) with a 210% increase in the risk of mortality compared to those who did not have triple-negative status. Alternatively, patients with Luminal B breast cancer (n = 34) reported the highest pooled mOS of 43.0 months (95% CI: 26.9–48.8). See Table 6.

### 3.7. Impact of Histology on mOS

In our analysis, the available data were insufficient to determine the mOS for specific histological types across all primary cancers, except for lung cancer. In the context of lung cancer, our findings indicate that patients with spinal metastases from small-cell lung cancer (SCLC) experienced a shorter pooled mOS of 6.3 months in contrast to those with NSCLC, who had a pooled mOS of 8.9 months. Notably, within the NSCLC subgroup, patients diagnosed with adenocarcinoma exhibited the most prolonged survival, with a pooled mOS of 25.3 months.

### 3.8. Impact of Spine Surgery on mOS

Only articles from the lung cancer primary site group contained adequate surgical intervention data for analysis. For lung cancer, three studies reporting on the mOS showed a weighted mOS of 14.2 months (95% CI: 4.9–18.5) for patients who received spine surgery, and a weighted mOS of 5.5 months (95% CI: 4.5–16.7) for patients who were not operated on [15,32,34]. The pooled HR for the two studies that reported on patients with lung cancer receiving spine surgery was 0.716 (95% CI: 0.48–1.06, *p* = 0.0992) [20,31].

## 4. Discussion

Spinal metastases represent 50% of all cancer-related bone metastases, with an increasing number of patients being affected due to comprehensive advances in oncological treatments that extend life expectancy, thereby providing a longer timeframe for spinal metastases to develop [48,49]. Breast and lung cancers are the most common malignancies that metastasize to the spine, accounting for 21% and 19% of cases, respectively [50]. The literature shows that 40% to 50% of patients with lung cancer develop bone metastases, with the spine involved in 63% of these cases [51]. Our study’s demographics highlight that lung cancer was the most common primary cancer type among the included studies, with a notable predominance of NSCLC subtypes. In our analysis of spinal metastases treatments, we demonstrate the notable advantage of novel treatments in improving the mOS, with targeted therapy for patients with lung cancer increasing the mOS by 15.7 months in contrast to more marginal gains with traditional modalities, such as chemotherapy, which extends survival by 5.7 months, and radiation therapy, which extends survival by 1.3 months in patients with lung cancer.

A comprehensive national survey found that 64.4% of lung cancer patients were diagnosed with spinal metastases concurrently with their lung cancer diagnoses [52]. Pathologically, NSCLC, specifically adenocarcinoma, was the most common type associated with spinal metastasis, accounting for up to 70% of cases—this is a finding that was also reflected in our study’s histological analysis [36]. In a 2015 study, Goodwin et al. [53] analyzed 26 patients with NSCLC that had metastasized to the spinal column, uncovering an mOS duration of 3.5 months within this cohort. Their research incorporated a combination of surgical, chemotherapeutic, and radiological treatments [54]. Conversely, our systematic review of patients with lung cancer, predominantly encompassing the NSCLC subtype—which represents over 90% of the lung cancer subjects—demonstrated an extended mOS of 6.7 months, with survival times ranging from 4.8 to 11.6 months. The prognosis of SCLC is consistently poorer than that of NSCLC [55]. Despite including individuals with SCLC and various other lung cancer types, our findings indicated a prolonged pooled mOS, underscoring the potential impact of incorporating advanced therapeutic approaches such as immunotherapy and targeted therapy. Similarly, the mOS of 5.7 months identified by Sellin et al. [56] surpasses the 3.6 months reported by Gokaslan et al. [57] in their review of 133 metastatic spinal melanoma cases from the same institution over two decades earlier. This implies that there was an enhancement in survival rates over time at this institution, which is possibly attributable to advancements in targeted medical therapy and RT. An increasing percentage of patients can be identified with a targetable mutation and treated with TT [1]. Despite the heterogeneity of data in our review, a key finding was the exceptional efficacy of standalone TT use. For example, in lung cancer, TT demonstrated a lower hazard ratio (HR = 0.395) than that of chemotherapy (HR = 0.604) and bisphosphonate therapy (HR = 0.933), indicating a more substantial reduction in the mortality risk compared to other treatment types; this is a conclusion that was drawn with statistical significance from the aggregated data of several studies. The most used TT agents in the included studies were EGFR-TKIs (including gefitinib and erlotinib), mammalian target of rapamycin (mTOR) inhibitors, and anaplastic lymphoma kinase (ALK) inhibitors. While outside the scope of our study, a future network meta-analysis could determine which TT agent, with or without concurrent bisphosphonate or chemotherapy, provides the largest survival benefit to patients with lung cancer with spinal metastases. In a previous study, a network meta-analysis of randomized trials identified denosumab to be superior to zoledronic acid for improving the overall survival and delaying skeletal-related events in metastatic bone disease due to lung cancer [58]. While we were not able to calculate pooled HRs for other primary cancer types due to insufficient data, TT consistently showed the largest difference in pooled mOS between the treated patients and control groups across all evaluated treatments.

A study by Sugita et al. [59] corroborates our findings, proving that TT improved life expectancy in patients with spinal metastasis. However, TT did not significantly affect local tumor control compared to traditional treatment, highlighting that surgery and RT remain mainstays of treating spinal metastasis. Their study suggests that the benefits of TT might be overshadowed by surgical interventions and that TT may not significantly enhance local control beyond the effects of surgery [59]. Interestingly, our study, which was based on a much larger patient population from multiple centers, evaluated the efficacy of TT as an independent treatment modality for spinal metastases, a subject infrequently explored in existing research. This is particularly relevant given that the National Institute for Health and Clinical Excellence guidelines advocate the commencement of radiotherapy for spinal metastases within 24 h of diagnosis [60]. There was significant heterogeneity in our data, primarily due to the exclusion of studies that concentrated on combination therapies, which represent the conventional methodology in cancer treatment but underscores a notable void in the research concerning the efficacy of TT in the absence of simultaneous surgical or RT treatment interventions. Furthermore, although the use of TT is on the rise, there is a significant gap in the development of therapeutic agents that can specifically target cancer within the unique bone milieu. The current literature is limited in providing safety, efficacy, and overall response rate estimates for many of these new treatment agents [1], reinforcing the need for more focused research on the support of adjunct medical therapy. Similarly, many studies in the literature discuss treatments and prognostics through the lens of algorithms that currently exist; the NOMS (neurologic, oncologic, mechanical stability, and systemic disease) framework and Tokuhashi score were developed over a decade ago and do not incorporate newer genomic data [8,61]. Future research should focus on adapting these scores, similar to the approach used by Cal et al. [19] in their study on lung cancer metastases to the spine. In their research, they introduced a modified prognostic score that incorporated the use of targeted therapy and tumor markers, building upon the foundation of the revised Tokuhashi score, which assesses the necessity of surgical intervention in these patients. Recent studies in the literature increasingly recognize the influence of targeted therapies on patient outcomes, underscoring the dynamic progression within orthopaedic oncology. This shift necessitates the incorporation of such therapeutic advancements into diverse prognostic scoring frameworks with the goal of enhancing their predictive accuracy for patient outcomes and informing effective treatment plans. Moreover, as previously mentioned, patients with lung cancer with spinal metastases who receive targeted therapy, a new advanced type of treatment, see a median improvement in survival of 10 months more than the next most effective treatment modality (chemotherapy). It is evident that basing treatment options on algorithms that fail to incorporate patients’ genomic data, such as receptor statuses that directly reflect eligibility for targeted therapy agents, can lead to non-ideal treatment plans. Patients may have a limited number of surgical treatment options presented to them because of their surgical candidacies being based on the outputs of the current incomprehensive scoring systems.

Similar to previous studies [62], in our systematic review, the efficacy of immunotherapy varied by cancer type. In lung cancer, it significantly reduced the mortality risk. However, data for breast and renal cancers were inconclusive due to a lack of detailed outcomes. The melanoma studies show mixed results: combining immunotherapy with targeted therapy improved the outcomes, while immunotherapy alone increased the mortality risk in another study [30]. When comparing our findings to previous studies in the literature [63], among 128 patients with metastatic spinal lesions from a combination of various types of primary cancers (lung, breast, renal, prostate, and melanoma), the mOS did not significantly differ between patients treated with immunotherapy and those who did not receive it. However, among patients who received immunotherapy, the mOS was shorter when the patients received immunotherapy before RT versus after RT [63].

Combination therapy, which involves the integration of two or more therapeutic agents, represents a fundamental strategy in cancer treatment [64]. Our study, however, had a limitation: the assessment of treatment modalities was conducted in isolation due to a lack of comprehensive data necessary for conducting pooled analyses of combination therapies. Furthermore, the inconsistent data reporting among these studies illustrates the challenges in synthesizing comprehensive information in this field. For instance, many studies reported the mOS solely for treatment groups, neglecting to include comparable data for the control groups. A pooled mOS was computed only when studies provided survival data for both the treated and control groups, and this “pooled” aggregation was feasible only if data for more than one study were available. The absence of such fundamental data on survival in many of the included studies draws attention to the prevailing gaps in data publishing practices, limiting our ability to draw definitive evidence-based conclusions and highlighting the urgent need for more thorough and standardized reporting in future research. Lastly, our study’s findings are subject to selection bias, particularly due to the criteria used to determine patient eligibility for surgery and other forms of medical management. This bias arises because patients who are chosen for these interventions often have better overall health and prognosis, meaning our results may not fully represent the broader population of individuals with spinal metastases, especially those who are deemed weaker surgical candidates or those who are otherwise unsuitable for these forms of treatment. Furthermore, a significant limitation in our systematic review is the lack of clarity in the included studies regarding how they addressed the selection bias associated with patient eligibility for each of the studied therapeutic interventions.

## 5. Conclusions

Based on our study’s systematic review and pooled data analysis, targeted therapy, specifically in lung and breast cancers, demonstrated the most notable improvements in the mOS as well as a more significant reduction in the mortality risk compared to other treatment types. Despite the inherent challenges posed by the heterogeneity of the available literature and the scarcity of complete quantitative data, our study navigates these complexities to provide a coherent overview of the current advancements in the treatment of spine metastases. Through this analysis, our review also advocates for a unified approach in future research, with a particular emphasis on standardizing reporting practices to enhance the clarity of original studies and facilitate impact-generating future reviews. Furthermore, similar to Cal et al.’s [19] refinement of the revised Tokuhashi score for lung cancer spinal metastases, future research should expand to include all types of primary tumor spinal metastases, incorporating targeted therapy, tumor markers, and novel genomic data into prognostic scoring systems to increase prognostic accuracy and to allow clinicians to appropriately guide management. Our study enables a deeper understanding of the evolving landscape of spine metastases treatment and provides further guidance to surgeons to help quantify the risks and benefits of treatment options based on a multitude of patient factors.

## Figures and Tables

**Table 1:** Inclusion and exclusion criteria for systematic review.

Study Component	Inclusion	Exclusion
Participants	A pathology of spinal cord metastases secondary to lung cancer, breast cancer, bone cancer, GI cancer, prostate cancer, thyroid cancer, melanoma, or kidney cancer	A pathology of inflammation, infection, or trauma
Interventions and comparators	Patients undergoing surgery, radiation therapy, targeted therapy, chemotherapy treatment, bisphosphonate therapy, immunotherapy, or no treatment for their spinal cord metastases	
Outcomes	Life expectancy based on survivorship and prognostic factors specific to patients with spinal cord metastases	Does not include the outcome of interest
Study designs	Present an original article reporting a case series greater than 5 patients: RCTs, clinical case series	Case reports, reviews
Publication	English studies published in a peer-reviewed, PubMed-indexed journal after 2005	Abstracts, editorials, letters, and duplicate studies or repeat publications of the same patient group; non-English papers; and papers published before 2005

**Table 2:** Study demographics.

No.	Reference	Year of Publication	Study Period	Design	Multicenter	Primary Cancer	LoE
1	Amelot et al. [14]	2019	2014–2017	Prospective	Yes	Breast	IV
2	Amelot et al. [15]	2020	2014–2017	Prospective	Yes	Lung	IV
3	Bollen et al. [16]	2014	2005–2012	Retrospective	Yes	Breast	IV
4	Dohzono et al. [17]	2017	2009–2016	Retrospective	No	Lung	IV
5	Lei et al. [18]	2016	2005–2015	Retrospective	No	Lung	IV
6	Cai et al. [19]	2019	2010–2018	Retrospective	No	Lung	IV
7	Kumar et al. [20]	2018	2001–2012	Retrospective	No	Lung	IV
8	Kato et al. [21]	2021	1995–2017	Retrospective	No	Renal	IV
9	Dohzono et al. [22]	2020	2009–2017	Retrospective	Yes	Lung	IV
10	Komatsu et al. [23]	2012	2004–2009	Retrospective	No	Lung	IV
11	Lin et al. [24]	2020	2001–2011	Retrospective	No	Lung	IV
12	Nagata et al. [25]	2013	2007–2009	Retrospective	No	Lung	IV
13	Tang et al. [26]	2015	2002–2013	Retrospective	No	Lung	IV
14	Mannavola et al. [27]	2020	1984–2019	Retrospective	Yes	Melanoma	IV
15	Zhao et al. [28]	2018	2005–2015	Retrospective	No	Breast	IV
16	Park et al. [29]	2023	2011–2017	Retrospective	No	Lung	IV
17	Shankar et al. [30]	2017	2012–2015	Retrospective	No	Melanoma	IV
18	Zang et al. [31]	2019	2006–2017	Retrospective	No	Lung	IV
19	Tan et al. [32]	2016	2001–2012	Retrospective	No	Lung	III
20	Rabah et al. [33]	2022	2010–2020	Retrospective	Yes	Breast	IV
21	Yang et al. [34]	2018	2010–2016	Retrospective	No	Lung	IV
22	Gómez-León et al. [35]	2018	2006–2016	Retrospective	No	Melanoma	IV
23	Zhai et al. [36]	2022	2009–2020	Retrospective	No	Lung	IV
24	Tan et al. [37]	2018	2001–2012	Retrospective	No	Breast	III
25	Shankar et al. [38]	2020	2010–2017	Retrospective	No	Renal	IV
26	Park et al. [39]	2016	2009–2015	Retrospective	No	Renal	IV
27	Chen et al. [40]	2021	2009–2021	Retrospective	No	Renal	IV
28	Kato et al. [41]	2023	1992–2017	Retrospective	No	Thyroid	IV

**Table 3:** Summary of studies based on cancer site, treatment types, grouping in isolated treatments, median survival rates, Cox regression hazard ratios, and cohort survival data.

				Primary Cancer Location				
				Lung, n = 16	Breast, n = 5	Renal, n = 3	Melanoma, n = 3	Thyroid, n = 1
Treatment type								
	Bisphosphonate therapy [n]			2	1	0	1	1
		Isolated use [n]		2	1	0	1	1
			Participant number reported for both groups [n]	2	1	0	1	1
			Median OS or OS rate reported for both groups [n]	2	1	0	1	1
			Univariable Cox regression hazard ratio reported [n]	1	0	0	0	0
			Multivariable Cox regression hazard ratio reported [n]	2	0	0	1	0
	Chemotherapy [n]			11	2	0	2	0
		Isolated use [n]		11	2	0	2	0
			Participant number reported for both groups [n]	9	2	0	2	0
			Median OS or OS rate reported for both groups [n]	5	1	0	1	0
			Univariable Cox regression hazard ratio reported [n]	2	0	0	0	0
			Multivariable Cox regression hazard ratio reported [n]	3	1	0	0	0
	Immunotherapy [n]			1	1	2	2	0
		Isolated use [n]		1	1	2	2	0
			Participant number reported for both groups [n]	1	1	1	2	0
			Median OS or OS rate reported for both groups [n]	0	0	0	1	0
			Univariable Cox regression hazard ratio reported [n]	1	0	0	1	0
			Multivariable Cox regression hazard ratio reported [n]	0	0	0	0	0
	Radiation therapy [n]			11	5	1	1	1
		Isolated use [n]		11	5	1	1	1
			Participant number reported for both groups [n]	9	4	1	1	1
			Median OS or OS rate reported for both groups [n]	3	2	0	1	1
			Univariable Cox regression hazard ratio reported [n]	2	1	0	0	0
			Multivariable Cox regression hazard ratio reported [n]	1	0	0	1	0
	Targeted therapy [n]			15	3	3	3	1
		Isolated use [n]		15	3	2	3	1
			Participant number reported for both groups [n]	15	3	1	3	1
			Median OS or OS rate reported for both groups [n]	11	2	0	1	0
			Univariable Cox regression hazard ratio reported [n]	6	1	0	2	0
			Multivariable Cox regression hazard ratio reported [n]	10	1	0	1	0
Cohort median overall survival reported [n]				12	4	2	3	1

n, number of studies.

**Table 4:** Patient demographics of all studies based on primary cancer location.

	Lung	Breast	Renal	Melanoma	Thyroid
n	2137	591	167	321	22
Weighted mean age [years]	58.3	58.3	61.9	56.3	58.1
Histology [n]	NSCLC: 2020 SCLC: 104 Others: 13			Nodular: 98 SSM: 90 Acral: 10 Other or NR: 123	Follicular: 16 Papillary: 6
Male sex, n (%)	1299 (60.8)	3 (0.5)	124 (74.2)		3 (13.6)
Pooled mOS (95% CI) [months]	6.7 (4.8–11.6)	28.3 (18.0–159.8)	58.3 (23.6–100.0)	10.6 (3.9–18.0)	123.0 (NR)

NR, non-reported.

**Table 5:** Pooled median overall survival and hazard ratios across all primary cancer locations based on treatment types.

			Primary Cancer Location							
			Lung			Breast		Renal	Melanoma	Thyroid
			Patients [n]	Pooled Median OS (95% CI) [Months]	Pooled HR (95% CI), *p*-Value	Patients [n]	Pooled Median OS (95% CI) [Months]	Patients [n]	Patients [n]	Patients [n]
Treatment Type										
	Bisphosphonate therapy									
		Yes	105		0.933 (0.579–1.503), 0.776	59		0	119	13
		No	131		–	25		0	173	9
	Chemotherapy									
		Yes	598	14.2 (5.5–19.9)	0.604 (0.248–1.467), 0.265	205		0	24	0
		No	721	8.5 (4.6–11.0)	–	64		0	280	0
	Immunotherapy									
		Yes	23			14		23	122	0
		No	187			171		42	187	0
	Radiation therapy									
		Yes	740	13.5 (5.1–22.9)		254	45.8 (45.7–46.0)	2	108	16
		No	618	12.2 (6.5–15.2)		152	33.5 (29.0–39.6)	22	182	6
	Targeted therapy									
		Yes	717	21.4 (11.0–23.6)	0.395 (0.296–0.527), <0.0001	191	83.2 (53.0–94.2)	9	70	4
		No	1168	5.7 (4.0–10.9)	–	166	32.9 (25.0–54.3)	56	252	18

**Table 6:** Impact of receptor status on median OS and HR for lung and breast cancer.

				Patients [n]	Pooled Median OS (95% CI) [Months]	Pooled HR (95% CI), *p*-Value
Primary Cancer Location						
	Lung					
		EFGR				
			Yes	367	24.9 (23.9–25.3)	0.664 (0.510–0.865), 0.002
			No	467	8.8 (5.2–14.6)	–
	Breast					
		Luminal A		113	27.8 (22.5–35.6)	
		Luminal B		34	43.0 (26.9–48.8)	
		Basal (Triple- negative)		88	11.6 (5.5–17.3)	3.204 (1.175–8.738), 0.023
		HER2+		106	38.2 (20.9–74.5)	0.449 (0.187–1.083), 0.075
		ER+		162	37.0 (32.0–61.0)	0.323 (0.187–0.559), <0.0001
		PR+		140	37.7 (36.0–49.0)	

## Data Availability

The data from this study are available on the PubMed database.

## Supplementary Materials

The following supporting information can be downloaded at https://www.mdpi.com/article/10.3390/cancers16071425/s1, Figure S1: PRISMA flow diagram; Table S1: PRISMA 2020 checklist.

## Appendix A

Search strings used for data extraction:

1. (“melanoma”[MeSH Terms] OR “melanoma”[Text Word] OR “skin cancer”[Text Word] OR “skin tumor*”[Text Word] OR “skin tumour*”[Text Word]) AND (“molecular targeted therapy”[MeSH Terms] OR “targeted therap*”[Text Word] OR “CTLA 4”[Text Word] OR “anti-PD-1”[Text Word] OR BRAF[Text Word] OR E3[Text Word] OR MEK[Text Word]) AND (“spine”[MeSH Terms] OR “spine*”[Text Word] OR “spinal”[Text Word] OR “vertebra*”[Text Word] OR “column*”[Text Word] OR “coccyx”[Text Word] OR “sacrum*”[Text Word])
2. (“prostatic neoplasms”[MeSH Terms] OR “prostat* cancer”[TW] OR (prostate AND (tumor OR tumour OR cancer OR carcinoma OR “squamous cell carcinoma” OR scc OR “transitional cell carcinoma” OR tcc OR “small cell”))) AND (“spine”[MeSH Terms] OR “spine*”[Text Word] OR “spinal”[Text Word] OR “vertebra*”[Text Word] OR “column*”[Text Word] OR “coccyx”[Text Word] OR “sacrum*”[Text Word]) AND (“molecular targeted therapy”[MeSH Terms] OR ”androgen receptor antagonist” OR MET OR VEGFR2 OR EGFR OR “anti-CTLA4” OR “targeted therap*” OR PARP OR BRCA)
3. (“osteosarcoma” [MeSH Terms] OR (bone AND (tumor OR tumour OR cancer OR sarcoma OR osteosarcoma))) AND (“molecular targeted therapy”[MeSH Terms] OR (“targeted therap*” OR “kinase inhibitor” OR RANKL OR NTRK OR “neurotrophic receptor tyrosine kinase” OR “immune checkpoint inhibitors” OR “PD-1” OR “interferon-alpha” OR “IFN-α” OR “interferon-alpha-2b” OR “IFN-α2b”)) AND (“spine”[MeSH Terms] OR “spine*”[Text Word] OR “spinal”[Text Word] OR “vertebra*”[Text Word] OR “column*”[Text Word] OR “coccyx”[Text Word] OR “sacrum*”[Text Word])
4. (“Stomach neoplasms” [MeSH Terms] OR((gastric OR stomach) AND (tumor OR tumour OR cancer OR adenocarcinoma OR “gastrointestinal stromal tumor” OR GIST OR “neuroendocrine tumor” OR carcinoid OR lymphoma))) AND (“molecular targeted therapy”[MeSH Terms] OR (“targeted therap*” OR VEGF* OR HER2* OR “ER+” OR “MSI-H“ OR dMMR OR “anti-CLDN18.2” OR “endothelial growth factor receptor” OR EGFR OR “tyrosine receptor kinase” OR TRK OR TRKI OR “TRK inhibitor” OR NTRK)) AND (“spine”[MeSH Terms] OR “spine*”[Text Word] OR “spinal”[Text Word] OR “vertebra*”[Text Word] OR “column*”[Text Word] OR “coccyx”[Text Word] OR “sacrum*”[Text Word])
5. (“thyroid neoplasms” [MeSH Terms] OR (Thyroid AND (tumor OR tumour OR cancer OR carcinoma OR papillary OR follicular OR medullary OR anaplastic))) AND (“molecular targeted therapy”[MeSH Terms] OR (“targeted therap*” OR RET OR “kinase inhibitor” OR “protein kinase” OR RET OR NTRK OR BRAF OR MEK OR “multikinase inhibitor”)) AND (“spine”[MeSH Terms] OR “spine*”[Text Word] OR “spinal”[Text Word] OR “vertebra*”[Text Word] OR “column*”[Text Word] OR “coccyx”[Text Word] OR “sacrum*”[Text Word])
6. (“Breast neoplasms” [MeSH Terms] OR (breast AND (tumor OR tumour OR cancer OR carcinoma OR adenocarcinoma OR “squamous cell carcinoma” OR scc OR “large cell carcinoma” OR “small cell”))) AND (“molecular targeted therapy”[MeSH Terms] OR “ER+” OR HER2 OR “anti-VEGF” OR mTOR OR “cyclin-dependent kinase 4/6” OR “CDK4/6” OR CDK4 OR CDK6 OR TP53 OR “tumor protein 53” OR “tumour protein 53” OR “phosphatidylinositol-4,5-bisphosphate 3-kinase” OR PIK3CA OR “fibroblast growth factor receptor” OR FGFR OR “cyclin D1 gene” OR CCND1 OR AKT1 OR Src OR “phosphatase and tension homolog” OR PTEN OR KRAS OR “anaphase promoting complex” OR “APC” or “NF1” OR “neurofibromatosis 1” OR MAP2K4 OR MAP3K1 OR AKT2 OR uPA OR “PAI-1” OR “targeted therap*” OR “PR+” or “PR positive”) AND (“spine”[MeSH Terms] OR “spine*”[Text Word] OR “spinal”[Text Word] OR “vertebra*”[Text Word] OR “column*”[Text Word] OR “coccyx”[Text Word] OR “sacrum*”[Text Word])
7. (“Lung neoplasms” [MeSH Terms] OR (lung AND (tumor OR tumour OR cancer OR carcinoma OR adenocarcinoma OR “squamous cell” OR SCC OR “large cell” OR “small cell” OR “small-cell” OR “large-cell” OR “nonsquamous cell” OR NSCLC tumor OR tumour))) AND (“molecular targeted therapy”[MeSH Terms] OR (“endothelial growth factor receptor” OR EGFR OR “tyrosine kinase inhibitors” OR TKI OR “TK inhibitors” OR RTK OR “c-Met” OR ErbB OR HER2 Or HER4 OR “human epidermal growth factor receptor 2” OR VEGF OR “vascular endothelial growth factor” OR “targeted therap*” OR “immune checkpoint inhibitors” OR CTLA4 OR “cytotoxic T-lymphocyte associated protein 4” OR “Anti-programmed death 1” OR “anti-PD1” OR “anti-programmed death ligand 1” OR “anti-PD-L1” OR “echinoderm microtubule-associated protein-like 4-anaplastic lymphoma kinase” OR “EML4-ALK” OR ALK OR BRAF OR MAP2K OR “mitogen-activated protein kinase 2”)) AND (“spine”[MeSH Terms] OR “spine*”[Text Word] OR “spinal”[Text Word] OR “vertebra*”[Text Word] OR “column*”[Text Word] OR “coccyx”[Text Word] OR “sacrum*”[Text Word])
8. (“Kidney neoplasms” [MeSH Terms] OR ((renal OR kidney) AND (tumor OR tumour OR cancer OR cell OR sarcoma OR carcinoma OR RCC OR Wilm*))) AND (“molecular targeted therapy”[MeSH Terms] OR cytokines OR mTOR OR IL-2 OR RTK OR TKI OR mTOR OR VEGF or “interferon-alpha” OR “IFN-α” OR “anti-PD1” OR “anti-CTLA4” OR “targeted therap*”) AND (“spine”[MeSH Terms] OR “spine*”[Text Word] OR “spinal”[Text Word] OR “vertebra*”[Text Word] OR “column*”[Text Word] OR “coccyx”[Text Word])
