# Supplementary figures and images for: The Impact of Adjunct Medical Therapy on Survival after Spine Metastasis: A Systematic Review and Pooled Data Analysis
Source: Cancers (Basel). 2024 Apr 7;16(7):1425. doi: 10.3390/cancers16071425 (PMC11011004; doi:10.3390/cancers16071425)

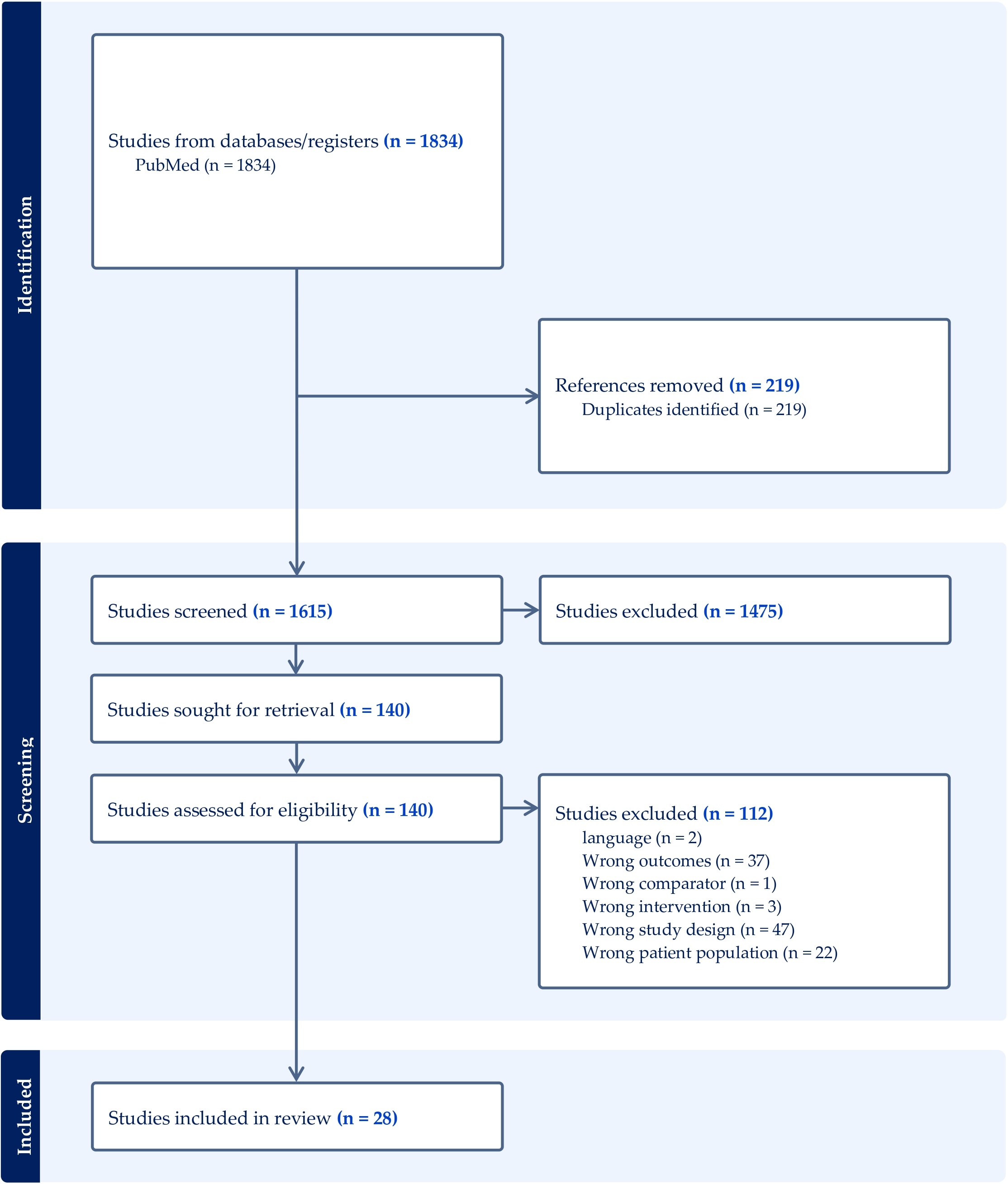

Supplement: Supplementary file 1 [file cancers-16-01425-s001.zip › PRISMA Flow Diagram.jpg]
